# Lutein and Zeaxanthin Content in 21 Plant Species from a Very Humid Premontane Forest in Colombia Palatable for Free-Range Laying Hens

**DOI:** 10.3390/plants12193484

**Published:** 2023-10-05

**Authors:** Yandy J. Aguillón-Páez, Gonzalo J. Díaz

**Affiliations:** Laboratorio de Toxicología, Facultad de Medicina Veterinaria y de Zootecnia, Universidad Nacional de Colombia, Bogotá 111321, Colombia; gjdiazg@unal.edu.co

**Keywords:** carotenoids, Colombia, forest, HPLC, plants, xanthophyll

## Abstract

Xanthophylls, such as lutein and zeaxanthin, have several functions in both plants and humans, including detoxification of oxidants (reactive oxygen species (ROS) and other radicals), maintenance of the structural and functional integrity of biological membranes, and photoprotection from intense light damage. The objective of the present study was to investigate the lutein and zeaxanthin content of 21 species of plants from a very humid premontane forest in Colombia during both dry and rainy seasons. The plants were selected based on being voluntarily eaten by laying hens under free-range conditions. Lutein and zeaxanthin were identified and quantified by high-performance liquid chromatography (HPLC). The results showed that all plants tested contained lutein, at levels ranging from 65.7 to 350 µg/g. Zeaxanthin levels were much lower (2.2 to 26.2 µg/lg) and were detected in only 5 of the 21 plants analyzed. Given that the lutein content of the plants tested in the present study was found to be comparable to that reported in marigold flowers (4–800 µg/g), it is possible that these plants can be used as a source of lutein in free-range laying hen production systems.

## 1. Introduction

Carotenoids are the second most abundant naturally occurring pigments on earth, being outnumbered only by chlorophyll. Carotenoid pigments are mainly C40 lipophilic isoprenoids and are synthesized by all photosynthetic organisms (bacteria, algae, and plants), as well as by some non-photosynthetic bacteria and fungi [1]. Carotenoids are known to play a key role in these processes due to their ability to deactivate triplet chlorophyll (^3^Chl^*^) and singlet oxygen (^1^O_2_^*^). Xanthophylls are also thought to be involved either directly or indirectly in the non-photochemical quenching (NPQ) of excess light energy in the photoprotector antenna of photosystem II [2]. On the other hand, carotenoids in leaves, flowers, and fruits attract insects for pollination and seed dispersal [3]. Two of the most abundant and important carotenoids are the xanthophylls lutein and zeaxanthin. Lutein is the most abundant plant carotenoid, and it has several important functions in the plant, including the following: (i) structural stabilization of antenna proteins, (ii) light harvesting (transfer of excitation energy to Chl), and (iii) quenching of ^3^Chl^*^ states. Zeaxanthin is an antioxidant in the lipid phase of the membrane, particularly under extreme high light stress, where it is important for plants to minimize photo-oxidative damage of membrane lipids [2].

Lutein and zeaxanthin also have an important role in preventing and reducing cataracts and age-related macular degeneration (ARMD) in humans [4]. Eggs are an important natural source of lutein and zeaxanthin because when these xanthophylls are present in the hen’s diet, they accumulate in the egg yolk [5]. This is the reason why commercial diets are often supplemented with these xanthophylls. The most important commercial source of lutein and zeaxanthin are the flowers of the “marigold” plant (*Tagetes erecta*), and extracts of these flowers are used to give a yellow color to the skin of commercial broilers and the eggs of laying hens [6]. However, due to the limited production and ever-growing price of marigold extracts, the search for alternative sources of xanthophylls for animal nutrition is important in order to fulfill consumers’ expectations in products such as chicken and eggs. Interestingly, more than 90% of the xanthophylls in marigold petal meal exist in the esterified form, whereas in alfalfa and corn, they are present as free alcohols [7].

Colombia has the second largest plant biodiversity in the world, with about 25,000 species from the 300,000 reported worldwide. Among these plants, it is highly likely to find some that might be rich in lutein and zeaxanthin, which in turn can be directly fed to poultry without the need for extraction and purification. The objective of the present study was to determine and quantitate the lutein and zeaxanthin content of the leaves of 21 plant species from a very humid premontane forest in Colombia during both dry and rainy seasons. The plants were selected on the basis of being voluntarily eaten by laying hens under free-range conditions.

## 2. Results

From the 21 plant species tested (Table 1), the highest lutein content was found in *Acalypha macrostachya* (Euphorbiaceae) in the July sampling (35 mg/100 g or 350 µg/g); this concentration was more than 5 times higher than the lowest concentration found (6.57 mg/100 g or 65.7 µg/g) for the April sampling in *Saccharum officinarum* (Poaceae).

The lutein content in *Trichantera gigantea* (Acanthaceae) in the November and July tests was lower (8.04 and 7.84 mg/100 g) than that found in the January and April samplings (10.3 and 10.1 mg/100 g, respectively). In *Xanthosoma sagittifolium* (Araceae), the lutein contents in the January and July samplings were higher (22.2 and 18.2 mg/100 g, respectively) than in the November and April samplings (15.7 and 15.2 mg/100 g, respectively).

In plants of the Asteraceae family, such as *Bidens rudifolia*, the November lutein levels were highest, and the July levels were lowest (16.5 and 8.3 mg/100 g, respectively), while the samplings from January and April showed similar concentrations (11.0 and 11.6 mg/100 g, respectively). The lutein content in *Melantera nivea* (also Asteraceae) was higher in the November sampling (24.9 mg/100 g) compared to the other three sampling times. The lowest lutein content was found in the April sampling (9.41 mg/100 g), while the samplings conducted in January and July showed intermediate concentrations (14.1 and 16.2 mg/100 g, respectively). On the other hand, the concentration of lutein in *Tithonia diversifolia* (Asteraceae) in November was low (8.75 mg/100 g) compared to the April content (17.6 mg/100 g). For the months of January and July, there was a difference of only 1.13 mg/100 g. In the case of *Sechium edule* (Cucurbitaceae), the lutein concentrations were similar across the four sampling times and ranged from 10.3 to 15.1 mg/100 g.

Two *Acalypha* spp. (Euphorbiaceae) were analyzed: *A. diversifolia* and *A. macrostachya.* In *A. diversifolia,* the November and January samplings showed the highest lutein content (28.2 and 23.3 mg/100 g, respectively), with lower levels found for the months of April and July (20.8 and 18.5 mg/100 g, respectively). *A. macrostachya* showed a higher lutein content in the two dry periods tested compared to the tests conducted during the rainy season. The lutein content for this plant was 32.0 and 35.0 mg/100 g for the months of January and July, compared to a lutein concentration of 18.9 and 21.8 mg/100 g for the months of November and April, respectively.

*Desmodium cajanifolium* (Fabaceae) showed the same pattern as the previously described *M. nivea* with a higher lutein content in the November sampling (31.4 mg/100 g) compared to the other sampling times (20.5, 14.3, and 20.8 for the samplings conducted in January, April, and July, respectively).

*Desmodium* sp. (Fabaceae) showed similar values for the tests conducted in rainy seasons (15.1 mg/100 g in November and 14.7 mg/100 g in April), while *Heliconia* sp. (Heliconiaceae) showed the highest lutein value in the July sampling (16.6 mg/100 g) and the lowest in the November sampling (8.7 mg/100 g); the January and April samplings showed intermediate values (10.3 and 13.2 mg/100 g, respectively).

In *Malachra rudis* (Malvaceae), the highest lutein content was found in the two rainy periods tested (16.4 and 22.0 mg/100 g for the months of November and April, respectively). The lutein concentrations in the months of January and July were 14.1 and 10.3 mg/100 g, respectively. *Sida poeppigiana* (Malvaceae) showed the highest lutein value in the July sampling (34.1 mg/100 g) and the lowest in the January sampling (17.9 mg/100 g).

Similar to the previously described *A. macrostachya*, *Musa paradisiaca* (Musaceae) showed a higher lutein content in the two dry periods tested compared to the tests conducted during the rainy season. The lutein content for this plant was 12.1 and 10.8 mg/100 g for the months of January and July, compared to a lutein concentration of 7.51 and 9.45 mg/100 g for the months of November and April, respectively. In the case of *Axonopus scoparius* grass (Poaceae), the lutein content ranged across the four sampling times, from 14.6 to 20.8 mg/100 g. The grass *Oplismenus burmannii* had a higher lutein content in July (21.4 mg/100 g) compared to November (12.3 mg/100 g), while the other Poaceae (*Zea mays*) showed the highest lutein content in April (17.9 mg/100 g) and the lowest in November (10.0 mg/100 g); the other two sampling times for *Zea mays* showed intermediate levels (10.9 and 11.8 mg/100 g for the January and July sampling, respectively).

*Solanum nigrescens* (Solanaceae) and *Myriocarpa stipitata* (Urticaceae) showed the highest lutein content in January (20.8 and 18.9 mg/100 g, respectively), while *Lantana camara* (Verbenaceae) showed higher lutein levels in the two rainy periods tested compared to the dry season. The lutein content for this plant was 14.3 and 15.7 mg/100 g for the samplings conducted in November and April, compared to a lutein concentration of 11.3 and 13.4 mg/100 g for the January and July samplings, respectively.

**Figure 1:**
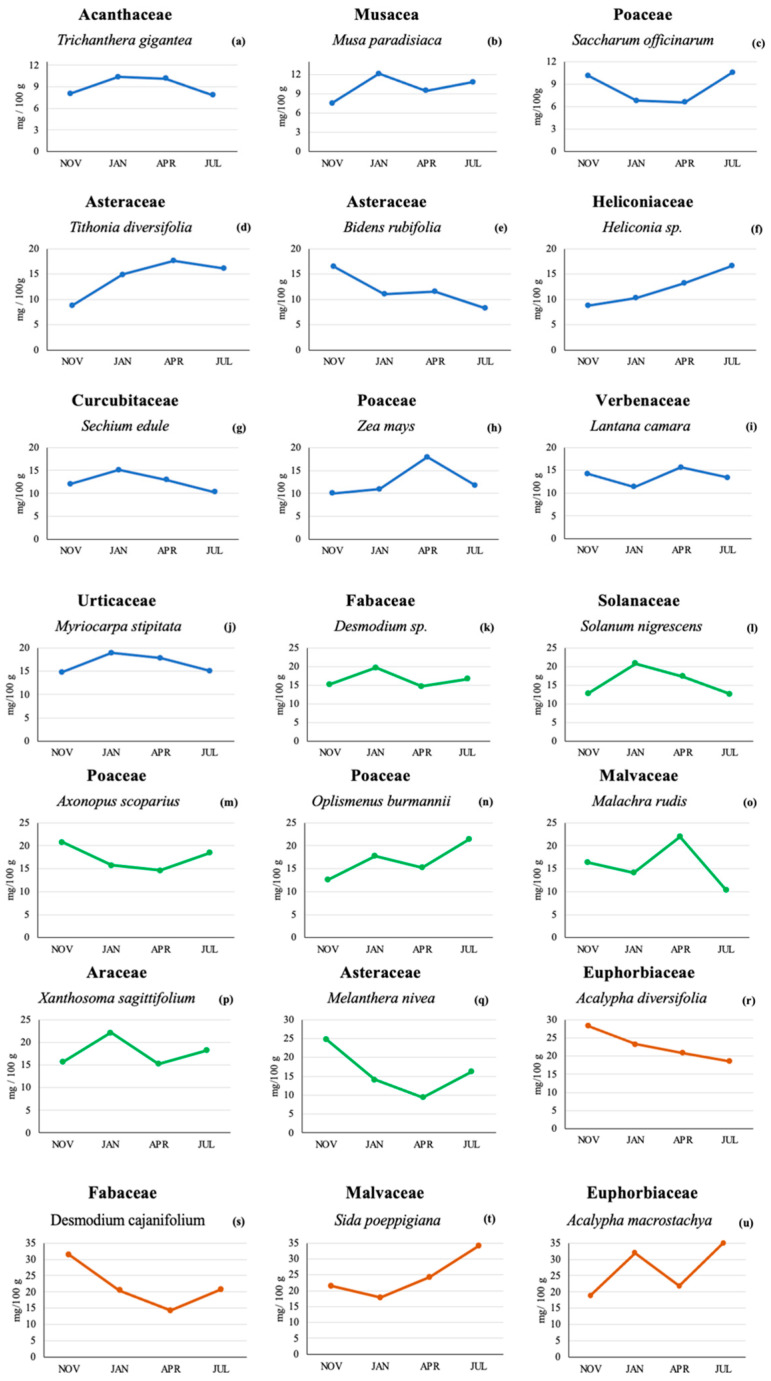
Lutein concentration (mg/100 g) by HPLC in 21 plant species from a Colombian very humid premontane forest sampled at four different sampling times. Lutein concentrations below 20 mg/100 g [line blue (**a**–**j**)], lutein concentrations below 25 mg/100 g [line green (**k**–**q**)] and lutein concentration levels below 35 mg/100 g [line orange (**r**–**u**)].

Figure 1 shows the lutein content found in the 21 plant species across the four sampling times. Lutein was found in all plants and at all sampling times, although the actual lutein concentrations varied among plants. A total of 10 plant species (Figure 1a–j) had maximum lutein concentrations below 20 mg/100 g. Seven plants had concentrations below 25 mg/100 g (Figure 1k–q), and 4 plants contained maximum levels below 35 mg/100 g (Figure 1r–u).

In regard to the other xanthophyll analyzed (zeaxanthin), it was only found in 5 of the 21 plant species analyzed (*D. cajanifolium, Desmodium* sp., *Z. mays, X. sagittifolium,* and *M. stipitate*) and at much lower concentrations than lutein. In the November sampling, *D. cajanifolium, Desmodium* sp., and *Z. mays* were found to contain zeaxanthin concentrations of 2.62, 1.21, and 0.91 mg/100 g, respectively. In January, none of the plants analyzed contained detectable levels of zeaxanthin. In the April sampling, only *X. sagittifolium* had detectable zeaxanthin levels (0.22 mg/100 g), while in July, only 2 species were found to contain detectable zeaxanthin levels: *X. sagittifolium* and *M. stipitata* (0.66 and 0.9 mg/100 g, respectively).

## 3. Discussion

Large differences in lutein content were observed within the same plant species across the different sampling times (typically, a difference of twice as much the concentration depending on the sampling time). It has been suggested that when plant leaves grow in extreme light environments (full sunshine or deep shade), they typically develop a set of morphological, physiological, and biochemical characteristics that optimize light capture and energy dissipation [8,9]. The most conspicuous morphological photoprotective mechanisms include chloroplast movements [10], changes in leaf orientation [11], increased thermal energy dissipation [12], or increased levels of antioxidants [13]. It might be possible that the differences in lutein content within the same plant observed in the present study could be related to differences in sun and shade patterns that the leaves received prior to sampling. These differences might represent the extremes of a light gradient in which leaves with differing potentials for light harvesting and dissipation are located within the canopy [14].

Previously published studies have shown differences in lutein accumulation in leaves due to differences in light exposure to plants. One of these studies examined summer–winter differences in photosynthesis, xanthophyll cycle-dependent energy dissipation, and antioxidant systems in populations of “*Mahonia repens* (Lindley) Don” growing in the eastern foothills of the Colorado Rocky Mountains in deep shade, full sun, and under a single-layer canopy of *Pinus ponderosa* (partially shaded) [15]. The lutein content per leaf area was significantly higher in summer than in winter. Another study investigated the effect of cultivar and season on carotenoids in lettuce (*Lactuca sativa*) and found that the carotenoid levels were higher in the summer [16]; ten anthocyanins were identified (cyanidins and delphinidins), while the main carotenoids found were all-trans-β-carotene (45–48%), lutein (13–20%), and zeaxanthin (11–15%). In other studies, the carotenoid content in New Zealand spinach was significantly higher in summer than in winter, and this was assumed to reflect seasonal variations rather than processing effects [17]. It is important to note that the above-mentioned studies were conducted in countries with marked daylight seasonal changes, whereas the present study was conducted in a tropical climate where daylight does not vary much between summer and winter (in an area located 5° North of the Equator). Near the Equator, the only differences observed during the year are in rainfall and cloudiness, not in daylight hours.

In regard to the large differences in lutein content found between different plant species, they could be due to the specific lutein accumulation characteristics of each particular species or to the different phenological stages at which the samples were obtained, since not all samples could be collected at the same stage. In endive (*Cichorium* spp.) and lettuce, the carotenoid concentrations of mature leaves were two to four times greater than those of young leaves [17]; in contrast, the younger leaves of New Zealand spinach (*Spinacia oleracea*) had slightly higher carotenoid levels than the mature leaves. In a study conducted with avocado (*Persea americana*), young leaves, fully expanded leaves, and old leaves had xanthophyll pigment concentrations of 138.5, 112.9, and 239.1 mmol mol^−1^ chlorophyll, respectively [9]. In another study, the seasonal progression of photoprotection responses in different aged savin juniper plants (*Juniperus sabina*) under shade and sun conditions was evaluated [18]. The results showed that although lutein may play an important role in dissipating excess energy, there was also a large seasonal effect on its concentrations that appeared to have little to do with irradiance. The authors suggested that when studying the ecophysiology of lutein, the effects of age and season must be considered in addition to the prevailing light regime.

Regardless of the within and between plant variations in lutein content found in the present study, it is important to note that the lutein content of the plants analyzed was comparable to that reported in marigold flowers. In the present trial, the lutein levels in leaves ranged from 6.57 to 35 mg/100 g, which is equivalent to 65.7 to 350 µg/g. These concentrations are comparable to those found in marigold flowers, in which lutein levels ranged from 4 µg/g in greenish yellow flowers to 800 µg/g in orange-brown flowers [19].

In contrast with lutein, zeaxanthin was only found in 5 of the 21 plants analyzed, and at much lower concentrations than lutein. A possible explanation for this finding is that plants produce zeaxanthin only under narrowly defined, specific environmental conditions, and exhibit rapidly fluctuating changes in its content, while the amount of lutein and β-carotene remain fairly stable [12]. In addition, it is possible that plants remove zeaxanthin when light availability is limiting to photosynthesis because zeaxanthin catalyzes the thermal dissipation of excess excitation [12]. Leaves exposed to such a fluctuating light environment form zeaxanthin in the morning and alternate rapidly between engagement and disengagement of existing zeaxanthin in the thermal dissipation of excitation energy. This strategy of the plant results in the retention of zeaxanthin in leaves during periods of low light by employing fluctuating light regimes under controlled conditions [12,20]. However, whatever the reason, this fact limits the availability of dietary zeaxanthin for plant eaters, either animals or humans [12].

In summary, the results of the present study show that even though all the plants tested (21 of 21) contained detectable levels of lutein, there were large differences in the ability of each plant species to accumulate lutein. Furthermore, the individual plant lutein content can vary depending on the time of the year. This suggests that plants behave differently depending on whether they are exposed to light intensities, as heavily shaded leaves do not have the ability to activate the xanthophyll cycle. Nonetheless, the lutein content of the plants tested was equivalent to that reported in marigold flowers, the “standard” for lutein used as a food and feed additive. The present study also found that the other xanthophyll analyzed (zeaxanthin) was present in only 23.8% of the plants evaluated and at much lower levels compared to lutein. More research is needed in order to investigate why there is such a difference in xanthophyll synthesis and accumulation.

Finally, since all the plants collected and analyzed correspond to plants that are voluntarily eaten by free-range laying hens, these plants can be used as a cheap source of lutein for the enrichment of egg yolks, given their importance in human eye health. More studies are needed in order to determine the potential use of these results to poultry production; for example, it would be interesting to investigate to what extent the plant lutein is transferred to the egg yolk and also the daily amount of each plant that a laying hen could eat without affecting laying production parameters.

## 4. Materials and Methods

### 4.1. Sample Material

**Figure 2:**
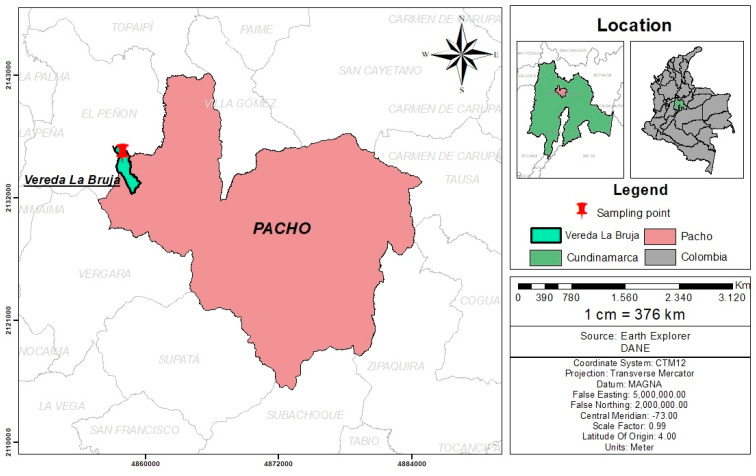
Geographical localization of the poultry farm where the sampling of the 21 plant species was carried out.

Sampling was carried out at a poultry farm located in county “La Bruja”, city of Pacho, Cundinamarca, Colombia (5°07′50″ North; 74°09′30″ West) (Figure 2). The area corresponds to a very humid premontane forest with a mean altitude of 1314 m and temperatures ranging from 14 to 25 °C all year long. All plants that were identified as palatable for free-range laying hens were collected for a total of 21 species. From each plant, a specimen was collected and sent for botanical classification to the Forest Herbarium “Gilberto Emilio Mahecha Vega”, Facultad del Medio Ambiente y Recursos Naturales of the Universidad Distrital “Francisco José de Caldas” in Bogota, Colombia. The species included in the study and their botanical names are summarized in Table 2.

For xanthophyll analysis, a total of four samples per plant were collected as follows: two during the “rainy season” (November 2021 and April 2022) and two during the “dry season”) January 2022 and July 2022). Sampling times were selected according to the historical records of rain and drought in the region. During sampling, approximately 500 g of leaves were collected from each plant, placed on newspaper, and taken to the laboratory (Laboratorio de Toxicología of the Universidad Nacional de Colombia) for the determination and quantitation of lutein and zeaxanthin, as described below.

### 4.2. Extraction of Carotenoids

Carotenoid extraction was performed according to a previously published method [21], with minor modifications. Table 3 summarizes the sample preparation procedure.

### 4.3. High Performance Liquid Chromatography (HPLC)

The chromatographic method was performed according to a previously published method [22], with minor modifications. Lutein and zeaxanthin were separated on a Phenomenex Develosil 5 µm RP-Aqueous C30, 140 Å, 250 × 4.6 mm I.D. analytical column, protected by a Phenomenex RP-C18 4 × 3.0 mm I.D. guard column (Phenomenex, Torrance, CA, USA), both kept at 16 °C. The separation was carried out using a gradient of two mobile phases at a flow rate of 1 mL/min, as follows: the starting composition was a mix of 90% mobile phase A (methanol:methyl-tert-butyl ether:1.5% ammonium acetate in water; 83:15:2, *v/v/v*) and 10% mobile phase B (methanol:methyl-tert-butyl ether:1.0% ammonium acetate in water; 8:90:2, *v/v/v*); this step was followed by a linear gradient from 10 to 45% B in 5 min, then a linear gradient from 45 to 95% B in 5 min, followed by 5 min at 95% B, after which the composition returned to the initial step (10% B) and was equilibrated for 10 min before the following injection.

**Figure 3:**
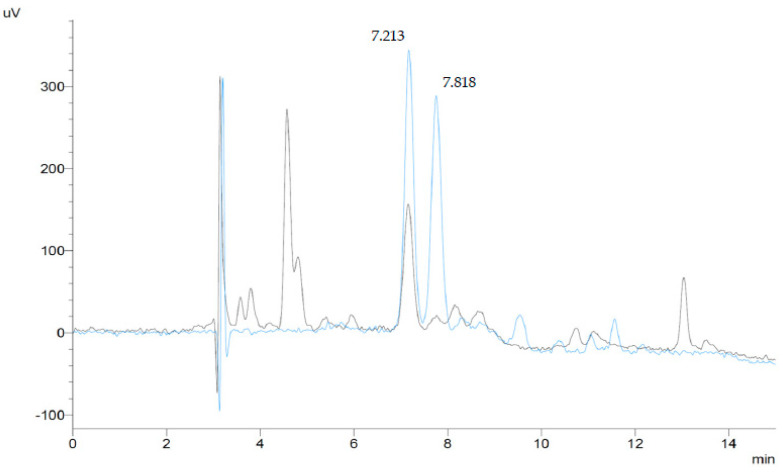
HPLC–chromatograms (445 nm) of a lutein and zeaxanthin standard mixture (0.1 μg/mL of each compound, blue line), and a plant sample extract (*Myriocarpa stipitata*) (black line). Peak 1 (tR = 7.213 min) corresponds to lutein, and peak 2 (tR = 7.818 min) corresponds to zeaxanthin.

HPLC analyses were conducted on a Shimadzu Prominence system (Shimadzu Scientific Instruments, Columbia, MD, USA) equipped with a DGU-20A3R degassing unit, two LC-20AD pumps, a SIL-20ACHT autosampler, a CTO-20A column oven, an SPD-20AV visible-ultraviolet spectrophotometric detector, and a CBM-20A bus module, all controlled by the Shimadzu “Lab Solutions” software. Absorbance was monitored at 445 nm for lutein and 450 nm for zeaxanthin, and the analytes were identified and quantified by means of external standards of known purity, prepared as described below. Figure 3 shows superimposed chromatograms of a standard mix of lutein and zeaxanthin and of a plant sample (*Myriocarpa stipitata*) containing both analytes.

### 4.4. Standard Solutions

Lutein and zeaxanthin are unstable and light sensitive, and all necessary precautions were taken to prevent their degradation prior to the analysis. Lutein and zeaxanthin standards were purchased from Fermentek Ltd. (Jerusalem, Israel). The lutein standard (Lot No. LU002) purity was 96.25%, whereas the zeaxanthin purity was 98.44% (Lot No. ZEA001). Stock standard solutions were prepared by weighing 2 mg of each xanthophyll, which were then dissolved in 25 mL tetrahydrofuran (THF) stabilized with 0.025% 2,6-di-tert-butyl-4-methylphenol (BHT) using a 25 mL amber volumetric flask. The stock solutions contained about 80 µg/mL of each xanthophyll.

### 4.5. Working Solution and Calibration Curve

A total of 5.0 µg of each analyte was taken from the stock solutions and diluted to 5 mL with stabilized THF in 7 mL amber vials; the final concentration was 1.0 µg/mL for each analyte. The calibration curve was prepared by pipetting 10, 20, 40, 80, or 100 µL of the working solution into 1.5 mL autosampler vials, to which 10 µL ascorbic acid in methanol were added as an antioxidant and taken to a final volume of 1.0 mL using mobile phase A as solvent. The linear regression equations for the lutein and zeaxanthin calibration curves were as follows: *y* = 70,372x − 135.72 for lutein and *y* = 71,573x − 95.848 for zeaxanthin. In both cases, the linear regressions had r^2^ values of 0.99.

### 4.6. Limit of Detection

The limit of detection (LOD) for lutein and zeaxanthin of the analytical technique was calculated based on the standard deviation of the response (S*y*) of the calibration curves and the slope of the calibration curve (S) at levels approximating the LOD according to the formula LOD = 3.3(S*y*/S) [23]. The limit of quantitation (LOQ) was calculated as three times the LOD. The calculated LOD and LOQ for lutein and zeaxanthin were 8 and 24 ng/mL in vials, respectively, and were identical for both compounds.

## Figures and Tables

**Table 1:** Lutein concentration (mg/100 g) by HPLC in 21 plant species collected in a very humid premontane forest in Colombia analyzed at four different sampling times.

Scientific Name	Family	Common Name (In Spanish)	November	January	April	July
**Season**			**Rainy**	**Dry**	**Rainy**	**Dry**
*Trichanthera gigantea*	Acanthaceae	Nacedero	8.04 ± 1.15	10.4 ± 0.26	10.1 ± 1.56	7.84 ± 0.41
*Xanthosoma sagittifolium*	Araceae	Bore	15.7 ± 0.14	22.2 ± 1.42	15.2 ± 1.95	18.2 ± 0.37
*Bidens rubifolia*	Asteraceae	Chipaca	16.5 ± 3.76	11.0 ± 0.60	11.6 ± 1.89	8.3 ± 5.62
*Melanthera nivea*	Asteraceae	Tamo-tamo	24.9 ± 0.51	14.1 ± 0.76	9.41 ± 0.51	16.2 ± 3.06
*Tithonia diversifolia*	Asteraceae	Botón de oro	8.75 ± 0.22	15.0 ± 1.14	17.6 ± 1.97	16.1 ± 2.74
*Sechium edule*	Cucurbitaceae	Guatila	12.1 ± 1.08	15.1 ± 1.30	13.0 ± 0.94	10.3 ± 4.17
*Acalypha diversifolia*	Euphorbiaceae	Suan	28.2 ± 0.50	23.3 ± 2.60	20.8 ± 2.57	18.5 ± 2.31
*Acalypha macrostachya*	Euphorbiaceae	Lechero	18.9 ± 0.55	32.0 ± 1.03	21.8 ± 1.85	35.0 ± 0.71
*Desmodium cajanifolium*	Fabaceae	Varilla	31.4 ± 1.83	20.5 ± 0.66	14.3 ± 1.09	20.8 ± 1.24
*Desmodium* sp.	Fabaceae	Pega-pega	15.1 ± 1.16	19.7 ± 0.11	14.7 ± 1.85	16.7 ± 0.65
*Heliconia* sp.	Heliconiaceae	Platanillo	8.7 ± 0.24	10.3 ± 1.47	13.2 ± 0.62	16.6 ± 0.29
*Malachra rudis*	Malvaceae	Malva	16.4 ± 1.35	14.1 ± 1.18	22.0 ± 4.75	10.3 ± 0.74
*Sida poeppigiana*	Malvaceae	Escoba	21.4 ± 0.62	17.9 ± 2.77	24.3 ± 1.49	34.1 ± 10.2
*Musa paradisiaca*	Musaceae	Plátano	7.50 ± 0.27	12.1 ± 0.92	9.45 ± 0.72	10.8 ± 0.45
*Axonopus scoparius*	Poaceae	Micay	20.8 ± 0.97	15.7 ± 1.96	14.6 ± 0.28	18.4 ± 6.89
*Oplismenus burmannii*	Poaceae	Grama	12.6 ± 0.11	17.7 ± 0.33	15.2 ± 1.36	21.4 ± 2.45
*Saccharum officinarum*	Poaceae	Caña de azúcar	10.1 ± 0.65	6.83 ± 0.11	6.57 ± 1.14	10.6 ± 2.13
*Zea mays*	Poaceae	Maíz	10.0 ± 0.42	10.9 ± 0.01	17.9 ± 0.94	11.8 ± 2.96
*Solanum nigrescens*	Solanaceae	Yerbamora	12.8 ± 0.02	20.8 ± 1.02	17.4 ± 1.47	12.6 ± 2.99
*Myriocarpa stipitata*	Urticaceae	Agüachente	14.8 ± 0.32	18.9 ± 3.18	17.8 ± 3.66	15.4 ± 1.43
*Lantana camara*	Verbenaceae	Venturosa	14.3 ± 0.65	11.3 ± 0.76	15.7 ± 1.01	13.4 ± 0.02

Values are means ± S.D. of duplicate analysis.

**Table 2:** Scientific name, botanical family, common name in Spanish, and phenological state of the collected plants.

Scientific Name	Family	Common Name (In Spanish)	Phenological State	Origin
*Trichanthera gigantea (Humbo & Bonpl)*	Acanthaceae	Nacedero	M	N
*Xanthosoma sagittifolium* (L.) *Schott*	Araceae	Bore	Vd	E/C
*Bidens rubifolia Kunth*	Asteraceae	Chipaca	B	N
*Melanthera nivea* (L.) *Small*	Asteraceae	Tamo-tamo	Vd	N
*Tithonia diversifolia (Hemsl.) A. Gray*	Asteraceae	Botón de oro	B	E/C
*Sechium edule (Jacq.) Sw.*	Cucurbitaceae	Guatila	Vd	E/C
*Acalypha diversifolia Jacq.*	Euphorbiaceae	Suan	Vd	N
*Acalypha macrostachya Müll. Arg.*	Euphorbiaceae	Lechero	Vd	N
*Desmodium cajanifolium (Kunth) DC.*	Fabaceae	Varilla	B	N
*Desmodium* sp.	Fabaceae	Pega-pega	B	N
*Heliconia* sp.	Heliconiaceae	Platanillo	I	N
*Malachra rudis Benth.*	Malvaceae	Malva	B	N
*Sida poeppigiana (K. Schum.) Fryxell*	Malvaceae	Escoba	B	N
*Musa paradisiaca* L.	Musaceae	Plátano	I	E/C
*Axonopus scoparius (Flüggé) Kuhlm.*	Poaceae	Micay	M	N
*Oplismenus burmannii (Retz.) P. Beauv.*	Poaceae	Grama	M	N
*Saccharum officinarum* L.	Poaceae	Caña de azúcar	Vd	E/C
*Zea mays*	Poaceae	Maíz	Vd	E/C
*Solanum nigrescens M. Martens & Galeotti*	Solanaceae	Yerbamora	B	N
*Myriocarpa stipitata Benth.*	Urticaceae	Agüachente	Vd	N
*Lantana camara* L.	Verbenaceae	Venturosa	B	N

Bloom: B; Inflorescence: I; Maturation: M; Vegetative development: Vd; Native: N; Exotic and Cultivated: E/C.

**Table 3:** Sample preparation for the determination of lutein and zeaxanthin in plant tissue (leaves).

1. Weigh 0.5 g of minced leaves in a 7 mL borosilicate culture tube with a Teflon-lined screw cap and add 5 mL of methanol. Homogenize in vortex stirrer. Leave at 4 °C overnight.
2. Remove from the refrigerator and centrifuge at room temperature at 1400× *g* (3000 rpm on a Hitachi centrifuge Model 05P-21) for 10 min; transfer the supernatant (methanol) to a 25 mL graduated flask.
3. Extract the pellet again with 5 mL tetrahydrofuran (THF), shake in vortex for 30 s, centrifuge at 1400× *g* for 10 min and combine the supernatant with the first extraction.
4. Repeat step 3 twice more.
5. Combine all supernatants in the 25 mL graduated flask and fill up to volume (25 mL) with THF.
6. Transfer 150 µL of the dilute extract into a 1.5 mL silanized autosampler vial and add 10 µL of ascorbic acid in methanol (1 mg/mL).
7. Add 1340 µL of mobile phase A for a final volume of 1.5 mL and homogenize in vortex.
8. Inject 10 µL into the liquid chromatograph.

## Data Availability

The data is contained within the manuscript. The manuscript raw data can be obtained in the following link: https://figshare.com/s/92655573a75ebbcc38fa, accessed on 16 March 2023.

## References

[1] Nisar N., Li L., Lu S., Khin N.C., Pogson B.J. (2015). Carotenoid metabolism in plants. Mol. Plant.

[2] Jahns P., Holzwarth A.R. (2012). The role of the xanthophyll cycle and of lutein in photoprotection of photosystem II. Biochim. Biophys. Acta Bioenerg. BBA—Bioenerg..

[3] Sandmann G., Schrader J., Bohlmann J. (2014). Carotenoids of biotechnological importance. Biotechnology of Isoprenoids.

[4] San Giovanni J.P., Chew E.Y., Clemons T.E., Ferris F.L., Gensler G., Lindblad A.S., Milton R.C., Seddon J.M., Sperduto R.D., Age-Related Eye Disease Study Research Group (2007). The relationship of dietary carotenoid and vitamin A, E, and C intake with age-related macular degeneration in a case-control study: AREDS Report No. 22. Arch. Ophthalmol..

[5] Zaheer K. (2017). Hen egg carotenoids (lutein and zeaxanthin) and nutritional impacts on human health: A review. CYTA J. Food.

[6] Alam A.U., Couch J.R., Creger C.R. (1968). The carotenoids of the marigold, *Tagetes erecta*. Can. J. Plant Sci..

[7] Quackenbush F.W., Miller S.L. (1972). Composition and analysis of the carotenoids in marigold petals. J. AOAC Int..

[8] Björkman O., Lange O.L., Nobel P.S., Osmond C.B., Ziegler H. (1981). Responses to Different Quantum Flux Densities. Physiological Plant Ecology I.

[9] García-Plazaola J.I., Matsubara S., Osmond C.B. (2007). The lutein epoxide cycle in higher plants: Its relationships to other xanthophyll cycles and possible functions. Funct. Plant Biol..

[10] Brugnoli E., Björkman O. (1992). Chloroplast movements in leaves: Influence on chlorophyll fluorescence and measurements of light-induced absorbance changes related to ΔpH and zeaxanthin formation. Photosynth. Res..

[11] Falster D.S., Westoby M. (2003). Leaf size and angle vary widely across species: What consequences for light interception?. New Phytol..

[12] Demmig-Adams B., López-Pozo M., Stewart J.J., Adams W.W. (2020). Zeaxanthin and lutein: Photoprotectors, anti-inflammatories, and brain food. Molecules.

[13] Grace S.C., Logan B.A. (1996). Acclimation of foliar antioxidant systems to growth irradiance in three broad-leaved evergreen species. Plant Physiol..

[14] Niinemets Ü., Bilger W., Kull O., Tenhunen J.D. (1998). Acclimation to high irradiance in temperate deciduous trees in the field: Changes in xanthophyll cycle pool size and in photosynthetic capacity along a canopy light gradient. Plant Cell Environ..

[15] Logan B.A., Grace S.C., Adams W.W., Demmig-Adams B. (1998). Seasonal differences in xanthophyll cycle characteristics and antioxidants in Mahonia repens growing in different light environments. Oecologia.

[16] De Souza A.S., de Oliveira Schmidt H., Pagno C., Rodrigues E., da Silva M.A., Flôres S.H., de Oliveira Rios A. (2022). Influence of cultivar and season on carotenoids and phenolic compounds from red lettuce influence of cultivar and season on lettuce. Food Res. Int..

[17] De Azevedo-Meleiro C.H., Rodriguez-Amaya D.B. (2005). Carotenoids of endive and New Zealand spinach as affected by maturity, season and minimal processing. J. Food Compost. Anal..

[18] Zhang J.L., Li X.G., Xu X.H., Chen H.P., Li Y.L., Guy R.D. (2021). Seasonal progression of photoprotection responses in different aged savin juniper plants under shade and sun. Trees.

[19] Gregory G.K., Chen T.S., Philip T. (1986). Quantitative analysis of lutein esters in marigold flowers (*Tagetes erecta*) by high performance liquid chromatography. J. Food Sci..

[20] Cohu C.M., Lombardi E., Adams W.W., Demmig-Adams B. (2014). Increased nutritional quality of plants for long-duration spaceflight missions through choice of plant variety and manipulation of growth conditions. Acta Astronaut..

[21] Perry A., Rasmussen H., Johnson E. (2009). Xanthophyll (lutein, zeaxanthin) content in fruits, vegetables and corn and egg products. J. Food Compost. Anal..

[22] Yeum K.J., Booth S.L., Sadowski J.A., Liu C., Tang G., Krinsky N.I., Russell R.M. (1996). Human plasma carotenoid response to the ingestion of controlled diets high in fruits and vegetables. Am. J. Clin. Nutr..

[23] Shrivastava A., Gupta V.B. (2011). Methods for the determination of limit of detection and limit of quantitation of the analytical methods. Chron. Young Sci..

